# Consensus Parameter: Research Methodologies to Evaluate Neurodevelopmental Effects of Pubertal Suppression in Transgender Youth

**DOI:** 10.1089/trgh.2020.0006

**Published:** 2020-12-11

**Authors:** Diane Chen, John F. Strang, Victoria D. Kolbuck, Stephen M. Rosenthal, Kim Wallen, Deborah P. Waber, Laurence Steinberg, Cheryl L. Sisk, Judith Ross, Tomas Paus, Sven C. Mueller, Margaret M. McCarthy, Paul E. Micevych, Carol L. Martin, Baudewijntje P.C. Kreukels, Lauren Kenworthy, Megan M. Herting, Agneta Herlitz, Ira R.J. Hebold Haraldsen, Ronald Dahl, Eveline A. Crone, Gordon J. Chelune, Sarah M. Burke, Sheri A. Berenbaum, Adriene M. Beltz, Julie Bakker, Lise Eliot, Eric Vilain, Gregory L. Wallace, Eric E. Nelson, Robert Garofalo

**Affiliations:** ^1^ Potocsnak Family Division of Adolescent and Young Adult Medicine, Ann & Robert H. Lurie Children's Hospital of Chicago, Chicago, Illinois, USA.; ^2^ Pritzker Department of Psychiatry and Behavioral Health, Ann & Robert H. Lurie Children's Hospital of Chicago, Chicago, Illinois, USA.; ^3^ Department of Psychiatry & Behavioral Sciences, Northwestern University Feinberg School of Medicine, Chicago, Illinois, USA.; ^4^ Department of Pediatrics, Northwestern University Feinberg School of Medicine, Chicago, Illinois, USA.; ^5^ Division of Neuropsychology, Children's National Medical Center, Washington, District of Columbia, USA.; ^6^ Center for Neuroscience, Children's Research Institute, Children's National Medical Center, Washington, District of Columbia, USA.; ^7^ Department of Pediatrics, George Washington University School of Medicine, Washington, District of Columbia, USA.; ^8^ Department of Neurology, George Washington University School of Medicine, Washington, District of Columbia, USA.; ^9^ Department of Psychiatry, George Washington University School of Medicine, Washington, District of Columbia, USA.; ^10^ Division of Endocrinology, Benioff Children's Hospital, University of California San Francisco, San Francisco, California, USA.; ^11^ Department of Psychology, Yerkes National Primate Research Center, Emory University, Atlanta, Georgia, USA.; ^12^ Department of Psychiatry, Boston Children's Hospital, Boston, Massachusetts, USA.; ^13^ Department of Psychiatry, Harvard Medical School, Boston, Massachusetts, USA.; ^14^ Department of Psychology, Temple University, Philadelphia, Pennsylvania, USA.; ^15^ Department of Psychology, Michigan State University, East Lansing, Michigan, USA.; ^16^ Nemours duPont Hospital for Children, Wilmington, Delaware, USA.; ^17^ Department of Pediatrics, Thomas Jefferson University, Philadelphia, Pennsylvania, USA.; ^18^ Bloorview Research Institute, Holland Bloorview Kids Rehabilitation Hospital, Toronto, Ontario, Canada.; ^19^ Department of Psychology, University of Toronto, Toronto, Ontario, Canada.; ^20^ Department of Psychiatry, University of Toronto, Toronto, Ontario, Canada.; ^21^ Department of Experimental Clinical and Health Psychology, Ghent University, Ghent, Belgium.; ^22^ Department of Personality, Psychological Assessment and Treatment, University of Deusto, Bilbao, Spain.; ^23^ Program in Neuroscience, Department of Pharmacology, University of Maryland School of Medicine, Baltimore, Maryland, USA; ^24^ David Geffen School of Medicine at UCLA, Los Angeles, California, USA.; ^25^ School of Social and Family Dynamics, Arizona State University, Tempe, Arizona, USA.; ^26^ Amsterdam UMC, Location VUmc, Department of Medical Psychology and Center of Expertise on Gender Dysphoria, Amsterdam, The Netherlands.; ^27^ Department of Preventive Medicine, University of Southern California, Los Angeles, California, USA.; ^28^ Department of Pediatrics, University of Southern California, Los Angeles, California, USA.; ^29^ Section of Psychology, Department of Clinical Neuroscience, Karolinska Institutet, Stockholm, Sweden.; ^30^ Centre for Cognitive Health in Brain Disease, Oslo University Hospital, Oslo, Norway.; ^31^ School of Public Health, University of California, Berkeley, Berkeley, California, USA.; ^32^ Department of Developmental and Educational Psychology, Brain and Development Research Center, Leiden University, Leiden, The Netherlands.; ^33^ Department of Neurology, University of Utah School of Medicine, Salt Lake City, Utah, USA.; ^34^ Department of Psychology, The Pennsylvania State University, University Park, Pennsylvania, USA.; ^35^ Department of Pediatrics, The Pennsylvania State University, University Park, Pennsylvania, USA.; ^36^ Department of Psychology, University of Michigan, Ann Arbor, Michigan, USA.; ^37^ GIGA Neurosciences, Liège University, Liège, Belgium.; ^38^ Department of Neuroscience, Rosalind Franklin University of Medicine & Science, Chicago, Illinois, USA.; ^39^ Center for Genetic Medicine Research, Children's National Medical Center, Washington, District of Columbia, USA.; ^40^ Department of Genomics and Precision Medicine, George Washington University, Washington, District of Columbia, USA.; ^41^ Epigenetics, Data, & Politics at Centre National de la Recherche Scientifique, Paris, France.; ^42^ Department of Speech, Language, and Hearing Science, George Washington University, Washington, District of Columbia, USA.; ^43^ Center for Biobehavioral Health, The Research Institute, Nationwide Children's Hospital, Columbus, Ohio, USA.; ^44^ Department of Pediatrics, The Ohio State University College of Medicine, Columbus, Ohio, USA.

**Keywords:** expert consensus, Delphi, puberty blockers, GnRHa, transgender, adolescents

## Abstract

**Purpose:** Pubertal suppression is standard of care for early pubertal transgender youth to prevent the development of undesired and distressing secondary sex characteristics incongruent with gender identity. Preliminary evidence suggests pubertal suppression improves mental health functioning. Given the widespread changes in brain and cognition that occur during puberty, a critical question is whether this treatment impacts neurodevelopment.

**Methods:** A Delphi consensus procedure engaged 24 international experts in neurodevelopment, gender development, puberty/adolescence, neuroendocrinology, and statistics/psychometrics to identify priority research methodologies to address the empirical question: is pubertal suppression treatment associated with real-world neurocognitive sequelae? Recommended study approaches reaching 80% consensus were included in the consensus parameter.

**Results:** The Delphi procedure identified 160 initial expert recommendations, 44 of which ultimately achieved consensus. Consensus study design elements include the following: a minimum of three measurement time points, pubertal staging at baseline, statistical modeling of sex in analyses, use of analytic approaches that account for heterogeneity, and use of multiple comparison groups to minimize the limitations of any one group. Consensus study comparison groups include untreated transgender youth matched on pubertal stage, cisgender (i.e., gender congruent) youth matched on pubertal stage, and an independent sample from a large-scale youth development database. The consensus domains for assessment includes: mental health, executive function/cognitive control, and social awareness/functioning.

**Conclusion:** An international interdisciplinary team of experts achieved consensus around primary methods and domains for assessing neurodevelopmental effects (i.e., benefits and/or difficulties) of pubertal suppression treatment in transgender youth.

## Introduction

Standards of care established by the World Professional Association for Transgender Health^1^ and the Endocrine Society^2^ recommend pubertal suppression for gender dysphoric transgender youth during early puberty (i.e., Tanner stages 2–3).^3,4^ Pubertal suppression is achieved through administration of gonadotropin-releasing hormone agonists (GnRHa). When administered in early puberty, GnRHa suppress endogenous sex hormone production and prevent the development of undesired and irreversible secondary sex characteristics, thereby minimizing distress associated with pubertal development incongruent with gender identity.^5^ For youth who later decide to initiate estrogen/testosterone (gender-affirming hormones [GAH]) treatment to induce development of the desired secondary sex characteristics, pubertal suppression may minimize the need for more invasive, surgical interventions (e.g., facial and chest surgery). For youth who decide not to pursue GAH treatment, discontinuing GnRHa will reactivate the hypothalamic-pituitary-gonadal axis and endogenous puberty will resume.^6^

Three longitudinal studies have examined psychosocial outcomes in GnRHa-treated transgender youth; two (conducted by the same research group) followed a single cohort over time, immediately before initiating GAH (*N*=70)^7^ and later in early adulthood after surgery for gender affirmation (*N*=55).^8^ The third study compared groups of GnRHa-treated (*n*=35) and untreated (*n*=36) youth longitudinally.^9^ Findings across these studies include significant reductions in depressive symptoms and improvement in overall psychosocial functioning in GnRHa-treated transgender youth. A fourth cross-sectional study compared adolescents diagnosed with gender dysphoria (GD), who were treated with GnRHa and close to starting GAH treatment (*n*=178), adolescents newly referred for GD evaluation (*n*=272), and cisgender adolescents recruited from the general population (*n*=651) on self-reported internalizing/externalizing problems, self-harm/suicidality, and peer relationships.^10^ Before medical treatment, clinic-referred adolescents reported more internalizing problems and self-harm/suicidality and poorer peer relationships compared to age-equivalent peers. GnRHa-treated transgender adolescents had fewer emotional and behavioral problems than clinic-referred, untreated adolescents and had comparable or better psychosocial functioning than same-age cisgender peers. In addition to studies of youth, the 2015 U.S. Transgender Survey included questions about past gender-affirming medical treatment, including pubertal suppression. These questions were asked retrospectively and linked to reported current and lifetime mental health.^11^ Individuals who received pubertal suppression treatment (*n*=89), when compared to those who wanted pubertal suppression, but did not receive it (*n*=3405), had lower odds of endorsing lifetime suicidal ideation on the survey. Given these five studies and the presumed reversibility of GnRHa treatment, pubertal suppression is increasingly offered to early pubertal transgender youth. It is important to note that there has been only one longitudinal report of adult outcomes,^8^ and questions remain regarding the potential for both positive *and* disruptive effects of pubertal suppression on neurodevelopment.^12–14^

The pubertal and adolescent period is associated with profound neurodevelopment, including trajectories of increasing capacities for abstraction and logical thinking,^15^ integrative thinking (e.g., consideration of multiple perspectives),^16,17^ and social thinking and competence.^18,19^ During this period, there is a developmental shift toward greater exploration and novelty seeking,^20,21^ salience of peer perspectives and interactions,^22^ and accelerated development of passions/interests and identities.^23^ These developments lay the groundwork for adult functioning.^18,24^ At the level of the brain, several primary neurodevelopmental processes unfold during adolescence, including myelin development^25^ and changes in neural connectivity^26^; synaptic pruning^27^ and gray matter maturation^28,29^; changes in functional connectivity^30^; and maturation of the prefrontal cortex^31^ and the “social brain” network.^19^ Adolescent neurodevelopmental processes underlie mental health risks, resilience, and outcomes.^32,33^

Considerable research has addressed the effects of puberty-related hormones on neurodevelopment, including hormone manipulation studies in nonhuman animals and observational studies in humans. Animal studies demonstrate pubertal hormones exert broad neuronal influence, including effects on neurogenesis, differentiation, apoptosis, dendritic branching, spine density, and regional gray and white matter volumes.^30,34^ Androgen and estrogen receptors are found in high density within the hypothalamus and amygdala, and are also present in the hippocampus, midbrain, cerebellum, and cerebral cortex of the rodent and monkey.^35–37^ This widespread receptor distribution in rodents may explain the diverse effects of pubertal hormones on both reproductive and nonreproductive behaviors, including anxiety, scent-marking, and food guarding.^34^ In human studies, pubertal progression has been linked to developmental changes in reward,^38^ social,^39^ and emotional processing^40^ as well as cognitive/emotional control.^41^ However, consensus regarding pubertal impacts at the neural level—such as puberty-associated changes observed in magnetic resonance imaging (MRI) measures—has been more difficult to achieve.^42^ Distinct puberty-related neurodevelopmental trajectories have been differentiated by sex.^43^

The combination of animal neurobehavioral research and human behavior studies supports the notion that puberty may be a *sensitive* period for brain organization:^44–46^ that is, a limited phase when developing neural connections are uniquely shaped by hormonal and experiential factors, with potentially lifelong consequences for cognitive and emotional health. Studies have linked early life adversity to early puberty onset^47^ and early puberty onset to poorer mental health.^48^ There is also some evidence to suggest that delayed puberty onset predicts slightly poorer adult functional outcomes.^49^ Taken as a whole, the existing knowledge about puberty and the brain raises the possibility that suppressing sex hormone production during this period could alter neurodevelopment in complex ways—not all of which may be beneficial.

Two small studies have assessed impacts of pubertal suppression on neural and cognitive functioning in peripubertal transgender youth. Staphorsius et al. compared brain and behavioral responses of GnRHa-treated (8 transgender girls [birth-assigned male] and 12 transgender boys [birth-assigned female]) and untreated transgender youth (10 of each sex) during an executive function task.^50^ No group differences were found in task load-related brain activation; GnRHa-treated transgender girls demonstrated poorer performance compared with untreated transgender boys and cisgender controls. Schneider et al. evaluated a single pubertal transgender girl undergoing GnRHa with MRI scans of white matter and cognitive assessments at baseline (before GnRHa initiation) and at 22 and 28 months of pubertal suppression treatment.^51^ During follow-up, white matter fractional anisotropy (i.e., a measure of axonal diameter, fiber coherence, and myelination) did not increase in the manner otherwise expected during puberty. By 22 months of pubertal suppression treatment, working memory scores dropped by more than half a standard deviation.

Larger-scale, longitudinal studies are required to understand possible neurodevelopmental impacts of pubertal suppression over time in transgender youth. Suppressing puberty may reduce dysphoria and diminish risks for poor mental health in this population, thereby exerting neuroprotective effects. If pubertal suppression disrupts aspects of neurodevelopment, it is possible these effects are temporary, with youth “catching up” developmentally after transitioning to GAH treatment or discontinuing GnRHa. However, pubertal suppression may prevent key aspects of development during a sensitive period of brain organization. Neurodevelopmental impacts might emerge over time, akin to the “late effects” cognitive findings associated with certain oncology treatments.^52^ In sum, GnRHa treatment might produce a myriad of *varied* impacts, both positive and disruptive.

The goal of this study was to develop a framework in which these questions could be asked, and ultimately answered. We identify priority research methodologies that can be used to address the empirical question of how pubertal suppression in transgender youth may affect neurodevelopment and real-world functioning. Given the complexity of neural development during the pubertal period and the novelty of developmental research with transgender youth, this study employed a Delphi consensus method to leverage international expertise in neurodevelopment, gender development, puberty/adolescence, neuroendocrinology, and statistics/psychometrics. By engaging a community of experts in an iterative consensus-building procedure, this study aimed to advance thinking about efficacious designs by moving beyond individual research efforts and single-discipline approaches.

## Methods

The Delphi procedure is a reliable iterative research method for establishing expert agreement,^53,54^ and has been used extensively to address health-related questions, particularly in emerging fields of clinical care.^55–57^ In the first round of a two-round Delphi procedure, a key question is presented to experts, who remain anonymous to one another throughout the Delphi process. Each expert provides responses/solutions to the question, which are then combined and organized by the study team. In the Delphi round two, experts rate each proposed statement/solution according to the level of agreement. Responses reaching the *a priori* consensus criterion are included as consensus statements. Given its anonymous iterative nature, the Delphi method avoids problems of typical expert work groups (e.g., adhering to the perspectives of more senior workgroup experts, inflexibly defending ideas) and allows for interaction among larger groups of experts from diverse locations and disciplines through asynchronous communication.^58–60^

We employed a two-round Delphi procedure to obtain expert consensus regarding the most efficacious research design elements to address the following research question: *What, if any, real-world impact does pubertal suppression have on transgender children's cognitive and neural development?* International experts in relevant research fields were identified and invited as follows:

1. An independent advisory panel consisting of five experts across key disciplines (see Acknowledgments section) was formed to identify international experts who, based on knowledge and experience, could best propose a research design to assess neurodevelopmental impacts of pubertal suppression in transgender youth.
2. Thirty-two recommended experts were vetted for their expertise; all met required criteria (i.e., a minimum of 10 first-author publications in relevant fields).
3. These experts were invited to participate in the Delphi procedure and were informed they would be invited to consider being a co-author of the resulting article. Twenty-eight experts responded: 20 agreed to participate, 4 declined due to lack of time, and 4 declined due to self-reported lack of expertise in this research area. Snowball sampling identified an additional 16 recommended experts, who were vetted (as described above) for their experience. Eight met criteria and were invited. Five of these experts participated, yielding a total of 25 experts agreeing to participate, 24 of whom completed the Delphi process. See Table 1 for academic institution locations and areas of expertise represented in the expert panel.

**Table 1.** Institutional Representation and Self-Reported Areas of Expertise

	*n*
Location of academic institution	
United States	16
The Netherlands	3
Belgium	2
Canada	1
Norway	1
Sweden	1
Self-endorsed areas of expertise^a^	
Brain development	13
Adolescent development	12
Neuroendocrinology	11
Neuroimaging	11
Neuropsychology	8
Cognitive development	7
Developmental assessment	4
Expert in GnRHa	2
Other (write in)	4
Developmental social neuroscience	1
Transgender health	1
Genetics of sex chromosomes	1
Gender development	1

^a^ Experts endorsed as many areas of expertise as applicable.

GnRHa, gonadotropin-releasing hormone agonists.

The Ann & Robert H. Lurie Children's Hospital of Chicago Institutional Review Board found that an expert Delphi consensus initiative did not require informed consent since the experts were direct partners in the research product. The first round of Delphi survey was distributed through the REDCap online survey platform and presented an overview of the research question with the following prompt for respondents: “What methods and tools should we use to identify clinically meaningful neurodevelopmental impacts of pubertal suppression? What type of longitudinal design and follow-ups are both practical and appropriate? What comparison groups might we consider?” This initial process yielded 131 distinct research design considerations; multiple descriptions of the same concept were collapsed into single statements. In the second Delphi round, each first-round research design consideration was presented back to the experts and rated as follows: a priority idea/approach or not a priority idea/approach. Experts could also select, “cannot rate due to lack of expertise.” The first Delphi round also yielded lists of potential comparison groups and assessment domains (29 items). In the second Delphi round, participants were asked to rank order these items according to priority. For the priority rankings of comparison groups, the top-rated comparison group by each expert was given a value of 2 and the second rated comparison group was given a value of 1. A mean was calculated for each comparison group option based on these values and these mean scores were used to identify the overall priority rankings. For the list of priority domains to measure, a parallel approach was taken with the top 6 domains ranked by each expert.

All experts participated in the second Delphi round. Twenty-two of the Delphi experts participated in the construction of the resulting article and are co-authors listed in reverse alphabetical order by last name (authors 5–26). The Results section contains the exact statements endorsed as a “priority” approach by 80% or more of the Delphi panel.

## Results

Four of the 131 individually presented statements were excluded from analyses because fewer than 15 experts rated them. Of the remaining 127 statements, 44 met the 80% or higher criterion for consensus and inclusion (see Table 2 for endorsement rates by statement). The average endorsement rate of included statements was 89.4%.

**Table 2.** Consensus Priority Recommendations Ordered by Consensus Ratings Within Categories

Study design considerations		
1	It would be helpful to follow these youth through and beyond initiation of cross-sex hormone treatment. Some aspects of human adolescent brain development are more related to pubertal hormone status than age *per se*, and to the extent that pubertal suppression may also put some features of brain development on hold; it would be good to know whether these features “catch up” once cross-sex hormone treatment has begun or whether a sensitive window for hormone-dependent brain development has closed.	22/22
2	Follow cohort after GnRHa treatment ends—collect data after the youth transition to GAH (when they complete their GnRHa treatment).	22/23
3	Any neurocognitive effect of GnRHa pubertal suppression may be complicated by the psychosocial and affective aspects of the transgender experience. This means that you would have to include multivariate models of both cognitive and psychosocial functioning.	22/23
4	Need to determine meaningful effect sizes and ensure sufficient statistical power for multiple expected comparisons with attrition.	21/22
5	Across the course of the study, three assessment points is the absolute minimum.	20/21
6	Need to use a multicenter design (not just one clinic).	21/23
7	Effects of GnRHa may not appear for several years. Any difference in brain structure due to GnRHa is likely to be seen over time (long term), rather than immediately.	20/22
8	Social and affective learning process may be affected by pausing puberty. These social and affective learning processes might cause subtle short-term differences that could ultimately cause clinically impactful and meaningful longer-term effects.	17/19
9	Of particular interest would be to also monitor the impact of hormonal therapy. One could then ask, “Does the trajectory change in response to cross-sex hormonal therapy or do they stay on the same trajectory as when they were on GnRHa?”	16/18
10	Assess target and comparison groups before puberty.	20/23
11	Need to manage the effects of repeated testing (i.e., minimize the practice effect of a longitudinal design with multiple time points).	19/22
12	The effect size will likely be small—therefore, you would need a large sample size.	19/23
13	The research design will need to account for the differences between youth who are assumed male versus assumed female as biological sex is differentially related to rate and pattern of cognitive development, connectome distinctiveness, and timing of peak brain volume.	19/23
14	All processes being studied (e.g., gender identity, mental health, and neural structure and function) display huge amounts of heterogeneity, and research methods that fail to capture this will provide distorted findings and lead to biased clinical recommendations. Analyses based on mean levels of these processes are unlikely to generalize to all individuals being treated (e.g., regressions or ANOVAs that compare groups with a slew of covariates). It is, therefore, necessary that enough data are collected per person to capture personalized trajectories of change across time. And the data need to be modeled in ways that reflect the heterogeneity of individual characteristics and trajectories.	18/22
Comparison groups and recruitment		
15	At least one control group should be cisgender participants as this area of research (i.e., hormones and the adolescent brain) is still rather new and more data are needed on all youth during this stage.	20/22
16	Critical to match the groups carefully to allow for evaluation of the effects of repeated testing (practice effects).	20/22
17	Comparison groups should be matched for pubertal stage.	19/21
18	Recruit all gender dysphoric youth across the pubertal age range, including those who are treated with GnRHa and those who are not.	18/21
19	This is not dissimilar from issues of discerning differences in cognitive trajectories in normal aging versus neurodegenerative disorders. The basic question involves cognitive growth curves among cisgender and transgender children overtime. There have been large-scale large-sample studies that have produced trajectories of brain development during the pre-pubertal, pubertal, and adolescent periods that could treated like a “brain growth curve.”	15/18
20	Need more than one comparison group to minimize the limitations of any one comparison group (no single comparison group is ideal).	18/22
Pubertal staging/measurement		
21	Measure gonadal hormone levels.	23/23
22	Collect information on menstrual cycle and contraceptive use for female adolescents involved in the study.	23/23
23	Measure Tanner staging (i.e., secondary sex characteristics).	21/23
24	Measure height/weight.	18/22
Domains to measure		
25	Use white matter microstructure scans (diffusion tensor imaging)—and use a longitudinal imaging pipeline (which exists) for processing these data with scientific rigor.	15/15
26	A pragmatic methodological implication is to consider: (1) not only relying solely on measures of performance and behavior but also measures of learning and motivation, and (2) not only relying solely on measures of cognitive capacities but also on social, affective, and value-based learning processes.	19/20
27	If MRI is included, consider imaging approaches focused on the following domains: social-emotional processing, executive functioning, risk and reward processing, and self-concept.	20/22
28	Studies in laboratory rodents show that testosterone, acting during puberty, programs the ability to adapt behavior as a function of social experience—therefore, include instruments that evaluate social proficiency.	19/21
29	Use diffusion tensor imaging to analyze white matter at the microstructural level.	17/19
30	Studies in laboratory rodents show that ovarian hormones, acting during puberty, program cognitive flexibility by exerting long-lasting effects on excitatory-inhibitory balance in prefrontal cortex—so include instruments that evaluate behavioral flexibility.	18/21
31	Examine white matter development, which is important for processing speed.	17/20
32	Important to measure emotional functioning because it is bidirectionally related to executive functioning.	16/19
33	Look at white matter characteristics since they seem to develop during puberty under the influence of sex hormones.	15/18
34	One cannot study everything or study everything well. It will be critical to identify the priorities in such a study, as there is a danger of doing too much here. Consider the outcomes that matter most and the hypothesized mediating mechanisms. Focus on the outcomes of interest.	19/23
35	There is no clear evidence that progressing through puberty later than peers is associated with delayed maturation of abstract reasoning, executive function, and social capacities.	18/22
36	Use structural MRI (T1/T2)—and use a longitudinal imaging pipeline (which exists)—for processing these data with scientific rigor.	13/16
37	There is an emerging shift in thinking about the increase in reward sensitivity and sensation-seeking during puberty as related to social value learning. Dopamine release is not primarily a “reward” signal, but rather a learning signal (e.g., prediction error signal)—the natural increased salience of social learning (status, prestige, being admired, respected, liked, etc.) These pubertal changes may have small effects on immediate behavior, yet that could contribute to changes in patterns of behavior over time, which could lead to large individual differences in developmental trajectories for people, such as if they had blocked puberty.	13/16
Measurement approaches		
38	In any study of cognitive change based on serial assessments, reliability of the measure is paramount. The consensus in the field is that tests should have a minimum test-retest reliability of >0.70.	20/20
39	Behavioral measurements should include standardized measures appropriate for repeated assessment with high test-retest reliability.	21/22
40	Match acquisition parameters between imaging sites.	17/18
41	Consider implementing measures and methods from large representative protocols, such as the ABCD.	17/18
42	Neuroimaging should parallel the behavioral study—neural measures should be linked to neurocognitive and behavioral measures.	19/22
43	For cognitive assessment, use a standardized battery for two reasons: (1) there might be a larger database of norms available that the cohort could be compared to, in addition to the likely to be small comparison (“control”) group, and (2) tasks within a category may be swapped in case of worries for learning effects.	18/21
44	Use “test batteries” that provide a general composite score as well as specific composites. By virtue of being composites, scores tend to be more reliable and stable than individual test scores.	17/20

The proportion represents the number of experts endorsing an item as a “priority” out of the total number of experts who rated the item as “priority” or “not priority.” The denominator represents the number of experts rating an item as a “priority” or “not priority” (as opposed to “cannot rate due to lack of expertise” or skipping the item).

ABCD, Adolescent Brain Cognitive Development Study; GAH, gender-affirming hormones; MRI, magnetic resonance imaging.

### Consensus parameter

#### Study design considerations

A multicenter design with more than a single clinic will be necessary to recruit a sufficient sample size, as the effect size will likely be small. Meaningful effect sizes must be determined to ensure sufficient recruitment to power multiple expected comparisons accounting for attrition in a longitudinal design. Three time points of measurement are the absolute minimum. It will be necessary to manage the effects of repeated testing with a particular focus on minimizing the practice effects of a longitudinal design with multiple time points. For cognitive assessments, standardized batteries should be employed as: (1) there may be a larger database of norms available that the cohort could be compared to, in addition to a local comparison (control) group(s), (2) general composite scores within test batteries tend to provide more reliable and stable scores than individual tests, and (3) tasks within a category may be swapped in case of worries for learning effects. In any study of cognitive change based on serial assessments, reliability of measures is paramount (the consensus in the field is that tests should have a minimum test-retest reliability of >0.70). It may be pragmatic to use measures and methods from large representative studies, such as the Adolescent Brain Cognitive Development (ABCD) Study.

All processes being studied (e.g., gender identity, mental health, neural structure, and function) display considerable heterogeneity, and methods that fail to capture this will provide distorted findings and lead to biased clinical recommendations. Analyses based on group means (e.g., regression or ANOVAs) are unlikely to generalize to all individuals being treated. Therefore, it is necessary to collect enough data per person to characterize individual trajectories of change over time.

Any GnRHa-induced neurocognitive effect may be complicated by psychosocial and affective aspects of the transgender experience. Therefore, multivariate models of both cognitive and psychosocial functioning should be included. Accounting for differences between birth-assigned male youth versus birth-assigned female youth is important, as sex is differentially related to the rate and pattern of cognitive development, connectome distinctiveness, and timing of peak brain volume. Assessments should begin before puberty in both treatment and comparison groups. The effects of pubertal suppression may not appear for several years. Any GnRHa-related difference in brain structure is likely to be observed over the long term, rather than immediately. Shifts in social and affective learning processes might cause subtle short-term differences that could ultimately result in clinically impactful longer-term effects. Therefore, studies should follow GnRHa-treated youth over time, including the time period after GnRHa treatment ends and/or when GAH commence. Some aspects of human adolescent brain development are more related to pubertal hormone status than age *per se*. To the extent that pubertal suppression may also put some features of brain development on hold, it is critical to know whether these features “catch up” (either once GAH treatment is initiated or if the adolescent elects to stop GnRHa and resume endogenous puberty), or whether a sensitive window for hormone-dependent brain development has closed. One way to measure this is to assess whether neurodevelopment shifts in response to initiating GAH following pubertal suppression: Do GnRHa-treated youth stay on the same neurodevelopmental trajectory as when puberty was suspended or does this trajectory change?

#### Comparison groups

To assess neurodevelopmental trajectories associated with GnRHa treatment, more than one comparison group is needed to minimize the limitations of any one comparison group. No single comparison group is ideal for this study question. A rank order of possible comparison groups is provided in Table 3. Groups should also be well matched, given the effects of a repeated testing design (e.g., practice effects). Matching for pubertal/developmental stage will be critical, including Tanner staging, gonadal hormone levels, height and weight, and, among youth assigned female at birth, menstrual cycle and contraceptive use. A primary comparison should be between GnRHa-treated transgender youth and untreated transgender youth, but it will also be important to include comparisons with cisgender samples as research on hormones and the adolescent brain is still novel and emerging and more data are needed on all youth during this developmental period. One way to accomplish the latter is to employ existing large-scale databases from studies of brain development during the pre-pubertal, pubertal, and later-adolescent periods, treating them as brain growth curves for comparisons. This approach is similar to the differentiation of cognitive trajectories in normal aging versus neurodegenerative disorders. The basic research question involves comparing cognitive growth curves over time.

**Table 3.** Rank Order of Priority Comparison Groups

Rank order of priority	Comparison group
1	Transgender youth who do not take GnRHa matched on pubertal status at the beginning of the study
2	Cisgender typically developing adolescents matched on pubertal status at the beginning of the study
3	Use a standardized battery and/or a large existing database of norms to compare to (in addition to a smaller comparison group)
4	Transgender youth who commence GnRHa treatment earlier compared to later in puberty
5	Siblings of transgender youth enrolling in the study (to serve as genetic and shared environmental controls)
6	Mixed clinical group of adolescents presenting for MH assessment/treatment in an outpatient setting matched on pubertal status
7^a^	Peers with mood disorders (to control for the overoccurrence of mental health distress in transgender youth) matched on pubertal status
7^a^	Youth with precocious puberty who are given GnRHa to delay puberty

This priority sequence was based on participants' top 2 ranked comparison groups, where the top rated comparison group was given a value of 2 and the second rated comparison group was given a value of 1. A mean score was derived for each comparison group based on participants' ratings and ordered from highest to lowest.

^a^ Comparison groups received the same mean score in the ranking.

#### Domains to assess

It will be critical to prioritize assessment domains based on hypothesized mediating mechanisms, with the most important domains to measure as follows: mental/behavioral health, pubertal stage, executive function/control, gender identity/dysphoria, and social awareness/functioning. See Table 4 for a complete list of ranked domains. Although we (the Delphi experts) identify executive function/control and social functioning as key domains to measure, it is important to note that there is no clear evidence that progressing through puberty later than peers is associated with delayed maturation of abstract reasoning, executive function, and social capacities. Executive function and emotional functioning are bidirectionally related, and for this reason, the two should be integrated in models/analyses. In addition, cognitive/behavioral flexibility, a component of executive functioning, should be measured, given that studies in rodents show ovarian hormones, acting during puberty, program cognitive flexibility by exerting long-lasting effects on excitatory-inhibitory balance in the prefrontal cortex.^61^ Studies in rodents also demonstrate that testosterone, acting during puberty, programs the ability to adapt behavior as a function of social experience.^34^ Measurement approaches should extend beyond cognitive capacities alone, embedding social, affective, and value-based learning processes. There is an emerging shift in thinking about increases in reward sensitivity and sensation-seeking during puberty as related to social-value learning.^18^ Dopamine release is not primarily a “reward” signal, but rather a learning signal (e.g., prediction error signal)—the natural increased salience of social learning (e.g., status and prestige, being admired, respected, and liked). The effects of suspending puberty on the salience of social-value learning might produce small near-term effects, but could contribute to changes in patterns of behavior over time, leading to large individual differences in developmental trajectories for GnRHa-treated youth.

**Table 4.** Rank Order of Priority Domains of Characterization and Assessment

Rank order of priority	Domains of characterization and assessment
1	Mental/behavioral health (including suicidality/hopelessness)
2	Pubertal stage/development (Tanner staging/hormone levels)
3	Executive function/control and attention
4	Gender identity/dysphoria
5	Social awareness/functioning
6	Quality of life
7	Brain/functional connectivity
8	Brain structure/volume
9	Emotional awareness/functioning
10	Physical health symptoms and outcomes (especially in adulthood)
11	Adaptive/independence skills
12	General cognitive functioning (IQ)
13	Sensation seeking, risk taking, reward sensitivity, and motivation
14	Genetics (i.e., possible impacts of GnRHa on DNA and RNA expression)
15	Academic functioning
16	Processing speed
17	Memory systems

This priority sequence was based on participants' top 6 ranked domains to measure, where the top rated domain was given a value of 6 and the second rated comparison group was given a value of 5, and so on. A mean score was derived for each domain based on participants' ratings and ordered from highest to lowest.

If neuroimaging is included, imaging approaches should focus on the following domains: social/emotional processing, executive functioning, risk and reward processing, and self-concept. Neuroimaging should parallel behavioral assessment. Neural measures should be linked to neurocognitive and behavioral measures. Acquisition parameters should be matched between imaging sites. Investigation of white matter development is important as myelination progresses during puberty, likely under the influence of sex hormones,^62^ and is related to cognitive processing speed. Both structural MRI and diffusion tensor imaging approaches should be used for white matter imaging and analyzed using a longitudinal imaging pipeline for processing these data with scientific rigor.

## Discussion

Puberty suppression has become an increasingly available option for transgender youth, and its benefits have been noted, particularly in the area of mental health. However, puberty is a major developmental process and the full consequences (both beneficial and adverse) of suppressing endogenous puberty are not yet understood. The experts who participated in this procedure believe the effects of pubertal suppression warrant further study, and this Delphi consensus process develops a framework from which future research endeavors can be built.

Expert consensus emphasized a minimum of three measurement time points, inclusion of multiple comparison groups to minimize the limitations of any one group, precision pubertal staging at baseline, accounting for sex in design and analysis, and the use of designs that capture heterogeneity in processes being studied. Focus on longer-term trajectories and outcomes was emphasized, given that effects of pubertal suppression on various processes may not be evident in the near term, and responses to delayed receipt of gonadal hormones may not be comparable to initial potentially organizing effects. Experts also highlighted that accounting for the psychosocial aspects of the transgender experience itself on development will require models that integrate both cognitive and psychosocial functioning. The highest endorsed measurement priorities were mental and behavioral health, executive function/cognitive control, and social awareness/functioning. The importance of interrelations between domains that mature during puberty/adolescence was also emphasized, including bidirectional relationships between cognitive and emotional control and links between reward sensitivity and social value learning. Regarding neuroimaging, experts stressed the importance of linking neural signatures to cognitive and behavioral measures, with attention to white matter development. Notably, while there was consensus in this approach to neuroimaging, there were divergent views as to whether a neuroimaging protocol should be prioritized in a study with limited resources. Some experts noted that insufficient work has been done on neural development during puberty in general and expending resources on an expensive neuroimaging protocol for this subset of youth may be premature, while others felt that defining underlying brain mechanisms by neuroimaging was important. Furthermore, at the final review of the article, four co-authors noted a concern with this specific Delphi consensus recommendation: “Accounting for differences between birth-assigned male youth versus birth-assigned female youth is important, as sex is differentially related to the rate and pattern of cognitive development, connectome distinctiveness, and timing of peak brain volume.” The four authors felt that instead of “peak brain volume,” a more appropriate measurement concept might be that of “structural brain metrics” (e.g., thickness and regional volumes).

Twelve different comparison groups were proposed in the first round of the Delphi and 8 of the 12 groups were rated as either first or second priority by at least 1 expert in the second Delphi round. This heterogeneity underscores the complexity of selecting comparison groups for this research and lends support to the experts' recommendation to engage more than one comparison group. The highest rated comparison groups were untreated transgender youth matched on pubertal stage, cisgender youth matched on pubertal stage, and a sample from a large-scale quasi-normative database (e.g., from the ABCD study) used as a “brain growth curve.” These comparison groups are not without weaknesses. Untreated transgender youth may differ in their intensity or experience of GD, level of parent support (e.g., are the parents against GnRHa treatment?), and socioeconomic status of the family and access to treatment (e.g., insurance coverage). A cisgender comparison group would lack gender-minority experience and associated stress.

Some statements approached, but did not reach consensus. For example, many experts suggested continuing assessments of transgender youth through young adulthood (mid-20s) when prefrontal development is near completion. Assessing adaptive functioning (everyday skills) over time due to the bidirectional link between executive functioning and adaptive behaviors was also often endorsed.

Not all relevant study considerations were raised by the Delphi panel. Neurodevelopmental impacts of pubertal suppression in transgender youth with neurodevelopmental differences/diagnoses (e.g., attention deficit/hyperactivity disorder and autism spectrum disorder) were not specifically addressed by the experts. Yet, evidence suggests an overoccurrence of neurodiversity characteristics (especially related to autism) among gender-referred youth.^55,63–66^ The neurodevelopmental impacts of pubertal suppression on neurodiverse gender-diverse youth might well be different than in neurotypical gender-diverse youth, given variations in neurodevelopmental trajectories observed across neurodevelopmental conditions.^67–69^

This study included experts from a range of relevant disciplines—a strength and also a possible limitation. The varied disciplines allowed for a broader range of ideas and perspectives, but some specialized recommendations might not have been sufficiently understood by Delphi experts from other disciplines. It is possible that some useful recommendations were lost in the process because few experts had backgrounds relevant to them. In fact, four recommendations were dropped from consideration because more than nine experts indicated they could not rate the item or skipped the item. These four items included topics related to advanced growth curve modeling, impact of GnRHa on immune system functioning, multifactorial relationships between GD and neurodevelopment, and challenges associated with using alternative forms of measures in longitudinal designs. The Delphi team included experts across the fields of neuroscience, neurodevelopment, developmental measurement, and gender development; however, most were not specialized in clinical transgender care *per se*. This reflects the dearth of transgender care clinicians/specialists with research productivity in adolescent neurodevelopment. Thus, the experts could comment with authority on neurodevelopment, including gender development/dysphoria aspects of study design, but the real-world clinical care considerations may well be underdeveloped in the proposed research design. For example, the everyday lived experience of transgender youth seeking gender-affirming medical care would be unfamiliar to most neurodevelopmental researchers. After the Delphi procedure was completed, one panelist commented that pubertal hormones might play a role in organizing neurodevelopmental gender-related trajectories, including identity itself, which would be important to consider for a developmental study of gender diverse youth.

Despite these limitations, an international expert team successfully completed an iterative Delphi procedure achieving consensus around priority research design elements to study neurodevelopmental impacts of pubertal suppression in transgender youth. The resulting consensus parameter addresses broad design issues, including comparison groups, longitudinal design, neurodevelopmental targets for assessment, and measurement approaches. While it may not be possible to incorporate all consensus methodologies into a single study, this parameter may serve as a roadmap for a range of research initiatives investigating pubertal suppression treatment in transgender youth.

## Notes

**Cite this article as:** Chen D, Strang JF, Kolbuck VD, Rosenthal SM, Wallen K, Waber DP, Steinberg L, Sisk CL, Ross J, Paus T, Mueller SC, McCarthy MM, Micevych PE, Martin CL, Kreukels BPC, Kenworthy L, Herting MM, Herlitz A, Hebold Haraldsen IRJ, Dahl R, Crone EA, Chelune GJ, Burke SM, Berenbaum SA, Beltz AM, Bakker J, Eliot L, Vilain E, Wallace GL, Nelson EE, Garofalo R (2020) Consensus parameter: research methodologies to evaluate neurodevelopmental effects of pubertal suppression in transgender youth, *Transgender Health* 5:4, 246–257, DOI: 10.1089/trgh.2020.0006.

## Abbreviations Used

ABCD: Adolescent Brain Cognitive Development
GAH: gender-affirming hormones
GD: gender dysphoria
GnRHa: gonadotropin-releasing hormone agonists
MRI: magnetic resonance imaging
